# A Phase 1 Two-Arm, Randomized, Double-Blind, Active-Controlled Study of Live, Oral Plasmid-Derived Adenovirus Type 4 and Type 7 Vaccines in Seronegative Adults

**DOI:** 10.3390/vaccines11061091

**Published:** 2023-06-12

**Authors:** Shannon Beaty, Natalie Collins, Nicos Karasavvas, Robert Kuschner, Jun Hang, Anima Adhikari, Irina Maljkovic Berry, Christian Fung, Shannon Walls, Elena Betancourt, Jason Mendy, Michael Lock, Emma Gierman, Sean Bennett, Paul Shabram, Kelly Warfield

**Affiliations:** 1 Emergent BioSolutions Inc., Gaithersburg, MD 20879, USA; 2 Walter Reed Army Institute of Research, Bethesda, MD 20814, USA; natalie.d.collins.mil@health.mil (N.C.);

**Keywords:** adenovirus, DNA virus, vaccine, clinical trial, mutation, preterminal protein

## Abstract

PXVX0047 is an investigational vaccine developed for active immunization to prevent febrile acute respiratory disease (ARD) caused by adenovirus serotypes 4 (Ad4) and 7 (Ad7). PXVX0047 consists of a modernized, plasmid-derived vaccine that was generated using a virus isolated from Wyeth Ad4 and Ad7 vaccine tablets. A phase 1 two-arm, randomized, double-blind, active-controlled study was conducted to evaluate the safety profile and immunogenicity of the investigational adenovirus vaccines. The two components of PXVX0047 were administered orally together in a single dose to 11 subjects. For comparison, three additional subjects received the Ad4/Ad7 vaccine that is currently in use by the US military. The results of this study show that the tolerability and immunogenicity of the PXVX0047 Ad7 component are comparable with that of the control Ad4/Ad7 vaccine; however, the immunogenicity of the PXVX0047 Ad4 component was lower than expected. Clinical trial number NCT03160339.

## 1. Introduction

Acute respiratory disease (ARD) caused by adenoviruses, most frequently by Adenovirus serotype 4 (Ad4) and serotype 7 (Ad7), is a concern in military training centers; Ad-associated ARD causes acute febrile illnesses that adversely impacts training, and incurs significant medical costs [1]. A live oral Ad4/Ad7 vaccine, initially reported by Chanock et al. in 1966 [2] and Top et al. in 1971 [3,4] and licensed by Wyeth in 1980 (“Wyeth Ad4/Ad7”), was successfully used to reduce the overall incidence of ARD in the military and eliminated the large, repeated outbreaks of adenovirus-associated ARD among military personnel that occurred prior to the advent of the vaccines. Wyeth ceased the production of Wyeth Ad4/Ad7 in 1994, resulting in the return of adenovirus-associated ARD outbreaks and occasional deaths in military recruit training facilities [5,6,7]. Barr Laboratories, now Teva Pharmaceuticals, restored the production of the Ad4/Ad7 vaccine using the original Wyeth Ad4 and Ad7 strains. This vaccine was observed to have a safety profile similar to placebo, with the expected shedding of the live virus in the stool for 28 days or longer after dosing [8,9]. The Food and Drug Administration (FDA) granted the licensure of the adenovirus type 4 and type 7 vaccine, live, oral (“Teva Ad4/Ad7”) in 2011 for use in military populations at risk of infection and it is currently administered to all new basic recruits in all the military services [10]. As anticipated, a sharp decrease in adenovirus-associated ARD was again seen following the reintroduction of the Teva Ad4/Ad7 vaccine in military recruits [11]. A post-licensure safety study of Teva Ad4/Ad7 in 100,000 recruits showed no statistically significant increase in any serious adverse events [12].

In response to a 2014 request for proposals by the US Department of Defense to modernize then production of the Ad4/Ad7 vaccine, PaxVax initiated the development of PXVX0047, a plasmid-derived vaccine that was generated using viruses isolated from Wyeth Ad4 and Ad7 vaccine tablets. PXVX0047 is an investigational vaccine developed for active immunization for the prevention of febrile ARD caused by Ad4 and Ad7 in the military population. This first study of PXVX0047 in humans was conducted to evaluate the safety profile and immunogenicity. The design of this study generally followed the design of the first-in-human study of Teva Ad4/Ad7 [9], except that it was only conducted in subjects who were seronegative for both Ad4 and Ad7. 

## 2. Results

The main criteria for the eligibility of the subjects were that the volunteers must be men or non-pregnant women between the ages of 18 and 35 years (inclusive) who were in good health and seronegative for both Ad4 and Ad7, as measured by both a luciferase-based and a colorimetric neutralization assay. Volunteers were recruited from the local civilian community at two different locations in the United States. A total of 124 volunteers were screened for this study; 110 subjects (89%) were excluded, most of whom (N = 88) were seropositive to either Ad4, Ad7, or both (Supplemental Figure S1, Supplemental Tables S3 and S4). Other reasons for screen failure included the withdrawal of consent (N = 6), sponsor terminating the study (N = 8), occupation which may create an increased risk of transmission of vaccine virus (N = 1), expected household contact which may create an increased risk of transmission of vaccine virus (N = 1), blood draw unsuccessful (N = 3), not using birth control (N = 1), subject age outside of the inclusion range (N = 1), or other health risks (N = 1). 

Among the 124 volunteers screened, a total of 14 were seronegative to both Ad4 and Ad7 by both screening assays and were enrolled in the vaccine groups in a 4:1 ratio: 11 subjects in the PXVX0047 group and 3 subjects in the Teva Ad4/Ad7 group. Among the 14 study subjects, the median age was 24.5 years, 50% were female, 50% were black or African American, 42.9% white, and 7.1% multiple races (Table 1). The demographics of the two treatment groups were similar. 

### 2.1. Vaccine Safety

In total, 8 of 11 PXVX0047 recipients and 3 of 3 Teva Ad4/Ad7 recipients reported at least one unsolicited adverse event (AE), which were all mild, moderate, or unrelated to vaccination (Table 2). Two severe unrelated AEs occurred that were found to be caused by food poisoning and animal allergy. Both treatment groups had similar laboratory results with no clinically relevant abnormalities. There were no safety-related study stops, severe vaccine-related adverse events, or vaccine-related serious adverse events (SAEs) or deaths.

Most solicited AEs in both groups were assessed as related to vaccination, though the small group sizes and the absence of a placebo arm prevents any analysis of causality and AE rates were similar in both treatment groups. All study-related solicited events were mild except one moderate headache. A total of 5 of 11 PXVX0047 recipients and 2 of 3 Teva Ad4/Ad7 recipients reported at least one related solicited AE from Days 1–8 during the confinement period, versus 3 of 11 and 1 of 3 during the post-confinement Days 8–15 (Supplemental Table S1). From Days 1 to 8, in PXVX0047 recipients, nasal congestion was the most common, reported in 4/11 subjects, and nausea, sore throat, vomiting, abdominal pain, chills, and myalgia were each reported in 1 subject. No fever or dyspnea was reported in either group. The median number of symptom days during the entire solicitation period (Days 1–15) was 2 days in PXVX0047 recipients and 3 days in Teva Ad4/Ad7 recipients.

### 2.2. Immunogenicity

**Figure 1:**
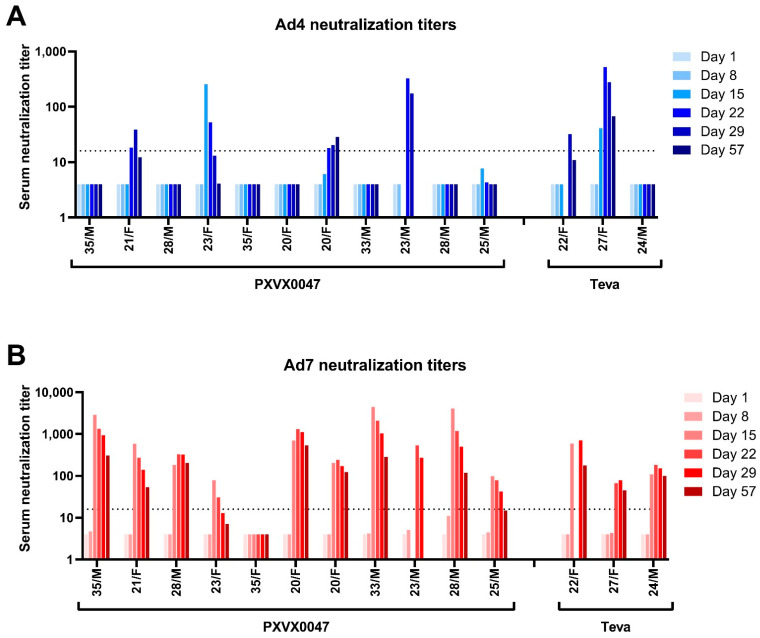
Ad4 and Ad7 neutralization titers, as measured by luciferase assay. Panel (**A**) shows results for the Ad4 vaccine and panel (**B**) shows results for the Ad7 vaccine. The age/sex of each study subject is shown on the horizontal axis. The groupings of subjects indicate the treatment with PXVX0047 or Teva. The dashed horizontal line indicates the threshold for seroconversion, defined as a 4-fold or greater rise from baseline. Neutralization data are not available for Teva 22/F Day 22 or 23/M Days 15 and 57.

Immunogenicity was evaluated by seroconversion as measured by the induction of 4-fold or greater rise from baseline anti-Ad4 and anti-Ad7 neutralizing activity. Two different assays were used to evaluate the neutralizing activity; the luciferase-based assay and the conventional colorimetric neutralization assay yielded similar seroconversion rates at all post-vaccination time points for both Ad4 and Ad7 (Figure 1, Supplemental Tables S7 and S8). Cumulatively, the Ad4 immune response was moderate, with 4 of 11 (36.4%) PXVX0047 recipients seroconverting by the Day 57 visit and 2 of 3 (66.7%) Teva recipients seroconverting by the Day 57 visit. In contrast, the immune response to Ad7 was markedly higher for both treatment groups, with cumulative seroconversion rates of 90.9% for PXVX0047 Ad7 (10 of 11 recipients) and 100% for Teva Ad7 (3 of 3 recipients). 

The geometric mean titer (GMT) of neutralizing antibodies to Ad4 and Ad7 was calculated at all post-vaccination time points using the luciferase-based assay and the colorimetric neutralization assay (Supplemental Table S5). The GMT of neutralizing antibodies to Ad4 remained low for subjects who received PXVX0047, with the peak GMT for the luciferase-based neutralization assay reaching 10.5 and the peak GMT for colorimetric neutralization assay reaching 10.0, while subjects who received Teva generated a higher GMT than in PXVX0047 recipients, which reached 34.8. Conversely, the GMT of neutralizing antibodies to Ad7 results revealed a robust response for subjects who received either the PXVX0047 or Teva vaccine, with the peak GMT for the luciferase-based neutralization assay reaching 391.9 and the peak GMT for the colorimetric neutralization assay reaching 115.6. 

### 2.3. Identification of a Mutation in the Ad4 Vaccine Strains at a Highly Conserved Amino Acid Position

The immunogenicity of the Ad4 component in the PXVX0047 vaccine was lower than previously reported for the Teva vaccine [8,9] and that observed for Teva subjects, although the treatment groups could not be compared using formal statistical tests due to the small size of the control group. We conducted additional studies into various potential causes of a low response rate to the Ad4 component in order to better understand the seroconversion findings in the clinical study. The sanger sequencing of the complete genome of the PXVX0047 Ad4 virus confirmed that it was identical to the published sequence of the virus obtained from Teva Ad4 vaccine tablets (GenBank accession #AY594254) [13] (Supplemental Table S6). Sequencing confirmation of both the PXVX0047 Ad4 virus and PXVX0047 Ad7 virus is described in detail in the test vaccine section of the Methods below. However, the further analysis of Ad4 sequences in GenBank revealed that, although the sequences of PXVX0047 and the Teva Ad4 vaccine viruses are identical to each other, they both differ from the wild-type Ad4 sequences in GenBank, and from the sequence that was used to generate the original Wyeth vaccine strain (strain CL68578; GenBank accession #AY458656) [14], by a single nucleotide substitution in the pre-terminal protein (pTP) at genomic nucleotide position 9171. While the PXVX0047 virus contains a thymine (T) at this position, the wild-type virus contains a guanine (G). This mutation results in a single amino acid difference at residue 388 of the preterminal protein (Teva and PXVX0047 = threonine (T); wild-type Ad4 = proline (P)).

**Figure 2:**
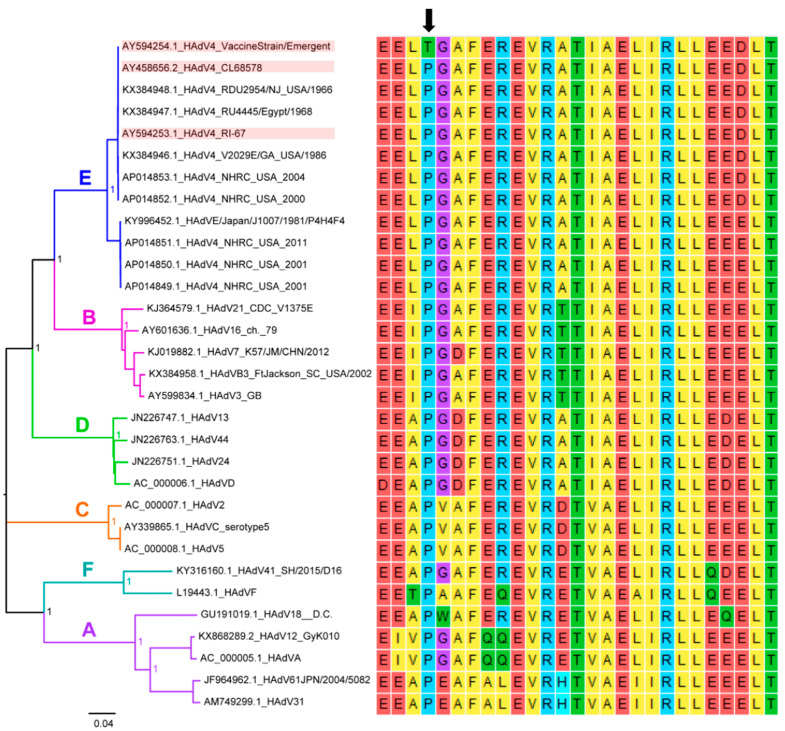
Nucleotide phylogenetic tree and the amino acid alignment of representative genomes of human adenovirus types. The PXVX0047 Ad4 vaccine strain (Genbank accession #AY594254.1), Ad4 vaccine reference strain CL68578 (Genbank accession # AY458656.2), and wild-type Ad4 reference strain RI-67 (Genbank accession #AY594253.1), are highlighted in red in the phylogenetic tree and amino acid alignment. The P388T residue change is shown in position 4 of the amino acid alignment, with a black arrow to annotate the location.

To determine whether this mutation exists in other human adenovirus types, complete and partial human adenovirus genome sequences (N = 252, human hosts only) were collected from GenBank and downloaded into an in-house database. The database includes six of the seven known types, A–F, consisting of 51 serotypes. Residue P388 of the preterminal protein was highly conserved in all human adenovirus types, and threonine was not found at this position in any of the wild-type viruses. The phylogenetic relationships and amino acid residues around P388 for human adenoviruses, including the vaccine strain, are shown in Figure 2.

## 3. Discussion

Currently, the FDA licensed Teva live oral Ad4/Ad7 is only utilized by the DoD to control ARD caused by Ad4 or Ad7 in military recruits. DoD sought to modernize the production of this live virus vaccine and partnered with Emergent BioSolutions (formerly PaxVax) to develop and clinically test PXVX0047, a plasmid-derived vaccine. As a vaccine platform, replicating the plasmid-derived viruses have the potential to reduce production/manufacturing cost significantly and eliminate the necessity of a seed-lot system while still stimulating a potent immune response. Additionally, plasmid-derived viruses can be leveraged as a vaccine vector, as in the case of Ad-vectored vaccines for HIV, influenza, and severe acute respiratory syndrome coronavirus 2 (SARS-CoV-2) [6,7,8].

We screened 124 young healthy individuals from the general U.S. population for Ad4 and Ad7 serostatus and demonstrated high rates of seropositivity for Ad4 and Ad7. A majority of the individuals in the screened population (89%) were seropositive for one or both of the adenoviruses tested and were ineligible to participate in the current study. This observed high seropositivity rate demonstrates the widespread circulation of these adenoviruses and these results are consistent with recent findings by Collins et al., showing high rates of adenovirus seropositivity among incoming military recruits [15]. Therefore, we can conclude that our study population is similar to those who volunteer for military service and give relevance to our findings.

A single oral dose of PXVX0047 was well tolerated. Consistent with the results for the currently licensed Teva Ad4/7 vaccine, the most commonly reported solicited AE was nasal congestion and all other noted solicited AEs were primarily mild in severity. Additionally, there were no abnormal vital signs, study halts, treatment-related serious adverse events, deaths, or other significant adverse events in either treatment group. Collectively, safety results from this study were consistent with the safety profile of the Teva Ad4/Ad7 vaccine [8,9] and support a positive outlook on the safety and tolerability of the PXVX0047 vaccine.

The anticipated peak shedding was Day 8; therefore, the Sanger sequencing of rectal swabs was performed for culture positive samples collected on Day 8 post vaccination. Interestingly, only one subtype, either Ad4 or Ad7 was detected in vaccine recipients and was not consistent with previous clinical trials; this may be attributed to technical failure from the self (volunteer)-collection of rectal swabs, and the improper handling/storage of samples or low virus recovery from the samples [5].

Results regarding both primary and secondary immunogenicity data showed that PXVX0047 yielded a robust immune response to Ad7 but elicited poor immunogenic responses to Ad4, as assessed by two distinct assay methodologies. The colorimetric-based neutralization assay is the conventional assay utilized in previous clinical trials to assess immunogenicity. We additionally tested samples using a luciferase-based neutralization assay developed for this study and the results were compared between the two methods for the confirmation of assay performance. The luciferase and colorimetric assays provided comparable results for the detection of serum-neutralizing antibodies to Ad4 and Ad7.

Nearly all subjects in both treatment groups met the Ad7 seroconversion definition, similarly to the expected rate of 93.8%, based on a Phase 3 clinical trial with the Teva live, oral Ad4/Ad7 vaccine [8]. The GMT of neutralizing antibodies to Ad7 results revealed a robust response, with the peak GMT for luciferase-based assay reaching 391.9 and the peak GMT for the colorimetric neutralization assay reaching 115.6. The Ad7 component of the PXVX0047 vaccine showed a robust immunogenic response and demonstrated that plasmid-derived viruses are suitable vaccine options that recapitulate the desirable safety and immune profiles of naturally occurring live virus vaccines. 

In contrast to Ad7, the immunogenicity results for the Ad4 component of PXVX0047 was markedly lower than the expected seroconversion rate of 94.5% [8]. At Day 29, four PXVX0047 recipients (36.4%) seroconverted for Ad4, while ten PXVX0047 recipients (90.9%) seroconverted for Ad7. The GMT of the neutralizing antibodies to Ad4 remained low for PXVX0047 (~10), as compared to the GMT for the Teva vaccine (34). Cumulatively, 4 of 11 (36.4%) PXVX0047 recipients had seroconverted by Day 57, while 2 of 3 (66.7%) Teva recipients had seroconverted by Day 57 visit; however, the small numbers in the groups prevent drawing comparative conclusions about the immunogenicity of the vaccines. The immunogenicity of the Teva Ad4/Ad7 vaccine should be confirmed in a larger number of volunteers to ensure it is maintaining effectiveness.

Subsequent investigation into the potential reasons for the lower immunogenicity of the Ad4 component of the PXVX0047 vaccine revealed that a single point mutation in the pTP gene appears to be responsible; all other factors related to manufacturing and administration were common to both Ad4 and Adv7 components of PXVX0047 and the weaker immune response was restricted to the Ad4 component alone. These unexpected results raised questions about how the P388T mutation in the pTP protein might account for the lower response rate, perhaps as the result of a defect in viral replication. Further investigation into this mutation revealed the novel finding that it reduces viral fitness in the gastrointestinal (GI) tract of human vaccinees, and confers a replication advantage in vitro [16]. While both Ad4 vaccines contain the mutant P388T strain, their genetic composition is not identical; the Teva vaccine was determined to contain a genetically mixed population comprising the mutant and wildtype, while the PXVX0047 vaccine is genetically homogenous and contains only the mutant strain. We believe that this genetic difference between Teva and PXVX0047 Ad4 vaccines primarily account for the observed differences in clinical outcomes.

In conclusion, the results of this phase 1 study demonstrate the tolerability and immunogenicity of PXVX0047 that are comparable with the data obtained using the Teva Ad4/Ad7 control vaccine for the Ad7 component. The immunogenicity of the Ad4 component of PXVX0047 was lower than anticipated and appears to be caused by a single point mutation in the pTP protein identified by Collins et al. that may have arisen during the development and/or manufacture of the licensed Teva Ad4 vaccine, from which the PXVX0047 vaccine was derived. Together, these data provide justification to continue with the clinical redevelopment of PXVX0047 Ad4 vaccine containing the wild-type (P388) pTP sequence.

## 4. Methods

### 4.1. Test Vaccine

PXVX0047 is a live Adenovirus Type 4 (Ad4)/Adenovirus Type 7 (Ad7) vaccine for single-dose oral administration. The Ad4 and Ad7 vaccines evaluated in this study were derived from military Ad4 and Ad7 vaccine tablets produced by Wyeth Pharmaceuticals, previously licensed by the FDA but beyond the expiration date. Adenovirus particles were grown from the Wyeth vaccine tablets and a viral stock was expanded from this material in A549 cells (human adenocarcinoma alveolar basal epithelial cells). Viral DNA was used to generate a cDNA plasmid encoding each of the respective viral genomes. 

Briefly, the construction of the recombinant vaccine plasmids was completed using established protocols for building large plasmid adenovirus cassettes through a combination of standard cloning and homologous recombination in bacteria. The published sequences of the Wyeth vaccines were used to design cloning primers that created overlap regions for recombination. The reference sequence used for Ad7 was GenBank accession #AY594256 [13] and the reference sequence for Ad4 was GenBank accession #AY594254 (Supplemental Table S6). These reference sequences were obtained from plaque-purified virus derived from Wyeth vaccine tablets. The entire viral plasmids were sequenced with at least two-fold coverage (GenBank accession #MN936177, Ad4; MN936178, Ad7) and compared against the published vaccine strain sequences. The Ad4 vaccine plasmid sequence was identical to the published Wyeth reference strain and the Ad7 plasmid shared a 99.96% identity with the published reference strain. For Ad7, eleven single nucleotide disagreements with the reference sequence were found in the genes Iva2, E2B, and L1; however, further investigation revealed that all 11 mutations were also found in the virus extracted from an Ad7 vaccine tablet vaccine from Teva Pharmaceuticals. Thus, both the Ad4 and Ad7 vaccine plasmid sequences were found to be identical to that of the virus extracted from military vaccine tablets.

Each of the recombinant vaccine virus stocks were produced in A549 cells by the transfection of the viral plasmid linearized by restriction enzyme digestion. The recovered virus was plaque-purified to ensure genetic homogeneity. To generate viral seed stocks, the recovered, plaque-purified virus was expanded in A549 suspension cells using serum-free media in disposable bioreactors. Cell-free supernatants were purified via size-exclusion and ion-exchange column chromatography steps prior to formulation in thermostable buffer and lyophilization. The sequencing of the viral seed stocks confirmed the absence of any mutations. The lyophilized viral seed material was formulated into aqueous-based, enteric coated capsules for use in this phase 1 clinical study. PXVX0047 was provided as a single oral dose of two enteric-coated capsules: one capsule of Ad4 and one capsule of Ad7. Each capsule contains no less than 32,000 (4.5 log10) 50% tissue-culture infective doses (TCID_50_) with lyophilization and bulking reagents. The placebo to match for PXVX0047 (PXVX047 PTM) was provided as a single dose of two enteric-coated capsules containing sucrose, which were indistinguishable in appearance from PXVX0047. 

### 4.2. Reference Vaccine

The reference vaccine used in this study for comparison was a live adenovirus type 4 (Ad4)/adenovirus type 7 (Ad7) vaccine manufactured by Teva Pharmaceutical Industries (Petah Tikva, Israel) for single-dose oral administration, which is licensed by the FDA for use in military populations. The Teva vaccine consists of unattenuated Ad4/Ad7 strains propagated in WI-38 human diploid fibroblast cells. Teva Ad4/Ad7 was provided as a single oral dose of two enteric-coated tablets: one white to off-white tablet of Ad4 and one light peach tablet of Ad7. Each tablet contains no less than 32,000 (4.5 log10) TCID_50_. The placebo to match for Teva Ad4/Ad7 (Teva PTM) was provided as a single dose of two commercially available ascorbic acid (Vitamin C) tablets similar in size, shape, and appearance to Teva Ad4/Ad7 tablets administered orally. These were not enteric-coated. 

### 4.3. Luciferase-Based Neutralization Assay

Ad4- and Ad7-specific neutralizing antibody titers in serum samples were determined using a luciferase-based neutralization assay using a similar methodology described previously [17] The assay is based on the capacity of antibodies to neutralize either recombinant Ad4-Luc or Ad7-Luc virus and quantified by the corresponding reduction in luciferase transgene expression. Serial two-fold dilutions (1:4 to 1:8192) of test sera were incubated with a pre-determined fixed concentration of Ad4-Luc or Ad7-Luc virus for 1 h at 37 °C followed by the addition of A549 cells. Following an 18 h incubation, luciferase activity is measured using the SteadyGlo Luciferase reagent system (Promega, Madison, WI, USA) and luminescence is quantified with a Spectomax M3 plate reader (Molecular Devices, Sunnyvale, CA, USA). Neutralization titers are defined as the maximum serum dilution that neutralizes 90% of luciferase activity and NT_90_ titers were determined using linear regression. The lower limit of quantitation (LLOQ) of the assay was 10.

### 4.4. Conventional Colorimetric-Based Neutralization Assay

In addition to utilizing the luciferase-based neutralization assay, the pre-vaccination serostatus and seroconversion of study subjects were determined utilizing the conventional colorimetric-based neutralization assay against Ad4 strain RI-67 and Ad7 strain 7a described previously by Collins et al. [15]. Briefly, heat-inactivated subject sera and controls were diluted 1:4 for the screening of vaccine subjects, 2-fold for human reference sera and positive rabbit reference sera, or 10-fold for negative rabbit sera and challenged with 200 TCID_50_ of either Ad4 or Ad7 in A549 cells. Following an incubation period of 7 days at 37 °C in 5% CO_2_, cells were visualized for cytopathic effect (CPE) utilizing neutral red and absorbance was read at 550 nm to measure the colorimetric CPE on cells. Neutralization at 1:4 indicated seropositivity. A similar method for seropositivity was followed to determine 50% neutralizing antibody titer (NT_50_) for assessing subject seroconversion; however, the subject sera was serially diluted two-fold from 1:4 to 1:8195 in PBS before challenge. The NT_50_ for subjects was determined by STATA.

### 4.5. Genetic Comparison of Ad4 Component of PXVX0047

An in-house database was created with complete and partial human adenovirus genome sequences screened from GenBank. Partial genome sequences were screened using the keywords, “Human adenovirus E2B” and “Human adenovirus pTP”. Although the E2B gene encodes both polymerase and pTP, the nucleotide residue is only found in pTP. Additionally, not all partial genomes had annotated serotype numbers, so the keywords ensured capturing all partial genomes possibly containing the mutation. The criteria for screening the complete genome sequences was (1) using the same keywords, “Adenovirus type [insert serotype number] complete genome” (i.e., “Adenovirus type 4 complete genome” searches for all complete human adenovirus E4 genomes); (2) the complete genome needs to have at least a serotype number; and (3) the length of the complete genome needed to be 30,000 to 35,000 bases long. Based on the criteria, the in-house database resulted in 252 complete or partial genomes that met criteria. The 252 in-house database genomes were aligned utilizing Geneious (version R10) and searched to find the corresponding nucleotide at residue 388 calling for proline (reference) or threonine (variant) in the E2B region of the genome. From the local database, 30 complete genomes were selected as representatives for A–F human adenovirus species and aligned using MAFFT and MEGAv7. The complete PXVX0047 Ad4 vaccine strain sequence was also added to the alignment. A neighbor joining phylogenetic tree was constructed to visualize the evolutionary relationships between the genomes and species. 

### 4.6. Selection of Study Population

The healthy adult male and female participants aged 18–35 were selected from the general population and represent an age group similar to that approved for the military use of the Teva vaccine. This choice of population enabled a robust preliminary assessment of the immunogenicity of PXVX0047 in a population not at risk of Ad4 or Ad7 ARD. Other inclusion criteria included: seronegativity for Ad4 and Ad7 by luciferase-based neutralization assay, using an acceptable method of contraception (if a female of childbearing potential or if male with female sexual partners of childbearing potential,), and able and willing to provide informed consent for study participation. Subjects with household, employment, or other contacts who are under the age of 7 years, pregnant, or immunocompromised were excluded to eliminate the possibility of the vaccine virus being shed in the GI tract of study subjects and spreading to vulnerable individuals. 

### 4.7. Study Design

This study (ClinicalTrials.gov identifier: NCT03160339) was conducted using a randomized, active-controlled, double-blind, double-dummy design with two treatment groups. Two study sites were used, which were located at the University of Vermont Medical Center in Burlington, Vermont and Cincinnati Children’s Hospital Medical Center in Cincinnati, Ohio. A total of 25 subjects were to be enrolled. This study consisted of a screening period of 60 days, an inpatient confinement and observation period from Day 1 to Day 8 in order to reduce the potential for the transmission of vaccine viruses, and an outpatient follow-up period through Day 57. During the outpatient follow-up period, subjects returned for visits at Days 15, 22, 29, and 57. A blinded safety review was conducted at Day 29 and data lock, unblinding, and final analysis were performed after all subjects had completed their Day 57 visit and all data queries were resolved. Eligible subjects were randomized to the PXVX0047 or Teva treatment groups in a 4:1 ratio. A single-dose vaccination was administered orally in each group on Day 1. To maintain blinding, each subject was also administered placebo capsules or tablets at the same time as the active vaccine. 

The sample size of this study was selected in order to enable initial the qualitative comparisons of safety and immunogenicity between treatment groups and historical data. Given a sample size of 20 PXVX0047 vaccine recipients, the chance of observing an uncommon AE—one expected to occur in only 1 in 10 vaccine recipients—at least once during the trial was calculated to be 88%. The sample size calculation assumed that PXVX0047 would be as immunogenic as the Teva Ad4/Ad7 vaccine with a seroconversion rate at Day 29 of about 94% for Ad4 and 94% for Ad7 [8]. Under these assumptions, power was determined to be 94%, which would result in the lower 95% confidence bound on both seroconversion rates to be 70% or higher. Similarly, it was determined that 78% power would result in a lower 95% confidence bound of 80% or higher. Given the final sample size of 11 PXVX0047 vaccine recipients, there was a 69% chance of observing an uncommon AE expected to occur in only 1 of 10 vaccine recipients. Under the original assumption that the PXVX0047 Ad4 and Ad7 seroconversion rates were both 94%, there was 74% power to indicate that the lower 95% confidence bound on both rates would simultaneously be 70% or higher.

### 4.8. Randomization

Prior to starting the study, the unblinded statistician created a randomization schedule comprising a list of treatment assignments—either PXVX0047 and Teva PTM or Teva Ad4/Ad7 and PXVX0047 PTM—in a random order. Treatments were assigned in a 4:1 ratio of PXVX0047 to Teva and allocated in permuted blocks of 5 subjects apiece. The schedule was then uploaded into the randomization module of the electronic data capture (EDC) system, Medrio. As subjects enrolled in the trial, after signing the informed consent form and confirmation of study eligibility, the EDC randomization module assigned the randomization number. Blinded study staff provided the randomization number to an unblinded pharmacist who matched the randomization number to a treatment allocation on the randomization schedule and dispensed the corresponding treatment to the blinded study staff. Subjects were considered enrolled once a randomization number had been assigned.

### 4.9. Safety Analysis

The primary safety objective was assessed through the evaluation of solicited AEs through Day 15, unsolicited AEs through Day 29, and the incidence of SAEs through Day 57. The percent of subjects who experienced a solicited AE was tallied by treatment group, broken out by the maximum severity reported and by relationship to treatment. The number of days that a subject experienced any solicited AE and each unsolicited AE was summarized using descriptive statistics and a frequency distribution. Given the small size of the control group, no formal statistical tests were used to compare the treatment groups.

The occurrence and severity of each of the 14 pre-defined AEs (abdominal pain, nausea, vomiting, diarrhea, cough, nasal congestion, dyspnea, sore throat, headache, fever, fatigue, chills, myalgia, and arthralgia) were solicited and recorded from each subject verbally from Day 1 through Day 8 and by diary card from Day 8 through Day 15. Subjects were counted only once at the highest severity reported. In contrast to solicited AEs, unsolicited AEs were reported spontaneously by the subject or discovered by the investigator. The number and percent of subjects who experienced an unsolicited AE through Day 29 were tallied by treatment group, system organ class (SOC), and preferred term (PT). All AEs were classified by SOC and PT according to the Medical Dictionary for Regulatory Activities (MedDRA) version 20.0. 

### 4.10. Specimen Collection and Immunogenicity Analysis

The primary immunogenicity analysis was based on the Ad4 and Ad7 seroconversion rates, measured independently of each other by the colorimetric-based assay, at Day 29, where seroconversion is defined as a 4-fold or greater rise in antibody titer over Day 1 (baseline). The seroconversion rate at each visit was quantified for Days 8, 15, 22, 29, and 57 for both the colorimetric-based and luciferase-based Ad4 and Ad7 neutralization assays. Colorimetric-based results reported as below the limit of the detection were set to 2, which is half the lower limit of quantitation (LLOQ) for the colorimetric-based neutralization assay. Luciferase-based neutralization assay titers below the limit of detection were set to 2.5, which is half the luciferase-based neutralization assay LLOQ, in calculations. Confidence intervals (CI) of 95% were calculated using the Wilson method [18].

Secondary immunogenicity analyses included estimates of the point and cumulative seroconversion rates through Days 8, 15, 22, 29, and 57 by both colorimetric-based and luciferase-based neutralization assays. Given the small size of the control group, no formal statistical tests were used to compare treatment groups. The cumulative Ad4 and Ad7 seroconversion rates through all post-vaccination time points were measured independently, where cumulative seroconversion through a particular visit is defined as having seroconverted at or prior to that visit. The geometric mean titer (GMT) of neutralizing antibodies to Ad4 and Ad7 at all post-vaccination time points was also measured independently. CIs for GMTs were calculated using normal-based methods to first construct a CI on log10-transformed data and then back-transforming the endpoints of the CI to the original data space.

### 4.11. Clinical Study Ethics

Ethical approval for the clinical study described here was granted by the Institutional Review Board (IRB) of PaxVax, Inc. (Redwood City, CA, USA) (now Emergent BioSolutions Inc.) and the U.S. Army Medical Research and Materiel Command Human Research Protection Office (HRPO) and the Walter Reed Army Institute of Research (WRAIR) Human Subjects Protection Branch (HSPB).

## Figures and Tables

**Table 1:** Demographics of the enrolled subjects. The age, sex, and race of subjects enrolled in each 1 treatment group are shown.

Characteristic	PXVX0047 N = 11	Teva Ad4/Ad7 N = 3	All Subjects N = 14
Age in y, median (range)	25 (20–35)	24 (22–27)	24.5 (20–35)
Sex, n (%)			
Female	5 (45.5)	2 (66.7)	7 (50.0)
Male	6 (54.5)	1 (33.3)	7 (50.0)
Race, n (%)			
Black/African American	6 (54.5)	1 (33.3)	7 (50.0)
White	4 (36.4)	2 (66.7)	6 (42.9)
Multiple	1 (9.1)	0	1 (7.1)
Other	0	0	0

**Table 2:** Overview of safety events. The number of subjects reporting at least one solicited or unsolicited adverse event are shown, along with the grade of the safety event.

Safety Event		PXVX0047 N = 11	Teva Ad4/Ad7 N = 3	All Subjects N = 14
**Subjects reporting at least one solicited adverse event**	Mild (grade 1)	1 (9.1%)	0	1 (7.1%)
	Moderate (grade 2)	3 (27.3%)	1 (33.3%)	4 (28.6%)
	Severe (grade 3)	1 (9.1%) *	0	1 (7.1%) *
	Potentially life-threatening (grade 4)	0	0	0
	**Total**	**5 (45.5%)**	**1 (33.3%)**	**6 (42.9%)**
Subjects reporting at least one unsolicited adverse event	Mild (grade 1)	5 (45.5%)	3 (100%)	8 (57.1%)
	Moderate (grade 2)	2 (18.2%)	0	2 (14.3%)
	Severe (grade 3)	1 (9.1%)*	0	1 (7.1%) *
	Potentially life-threatening (grade 4)	0	0	0
	**Total**	**8 (72.7%)**	**3 (100%)**	**11 (78.6%)**

* safety events found to be unrelated to vaccination.

## Data Availability

The data that support the findings of this study are available from the corresponding author upon request.

## Supplementary Materials

The following supporting information can be downloaded at: https://www.mdpi.com/article/10.3390/vaccines11061091/s1, Figure S1: Disposition of study subjects; Table S1: Treatment-related solicited adverse events by time period; Table S2: Seroconversion rates by timepoint, as measured by luciferase assay; Table S3: Ad4 and Ad7 serostatus at screening by luciferase assay; Table S4: Ad4 and Ad7 serostatus at screening by colorimetric assay; Table S5: Neutralizing antibodies by timepoint: Geometric mean titers; Table S6: Ad4 and Ad7 viral vaccine sequence strains; Table S7: Ad4 neutralization titers, as measured by luciferase assay; Table S8: Ad7 neutralization titers, as measured by luciferase assay; Figure S2: Ad7 neutralization titers, as measured by CPE assay; Table S9: Seroconversion rates by timepoint, a measured by colorimetric assay.
