# Supplementary material for: A Phase 1 Two-Arm, Randomized, Double-Blind, Active-Controlled Study of Live, Oral Plasmid-Derived Adenovirus Type 4 and Type 7 Vaccines in Seronegative Adults
Source: Vaccines (Basel). 2023 Jun 12;11(6):1091. doi: 10.3390/vaccines11061091 (PMC10301169; doi:10.3390/vaccines11061091)
Supplement: Supplementary file 1 [file vaccines-11-01091-s001.zip › vaccines-2422090-supplementary.pdf]

SUPPLEMENTARY INFORMATION

Supplemental Figure S1. Disposition of Study Subjects

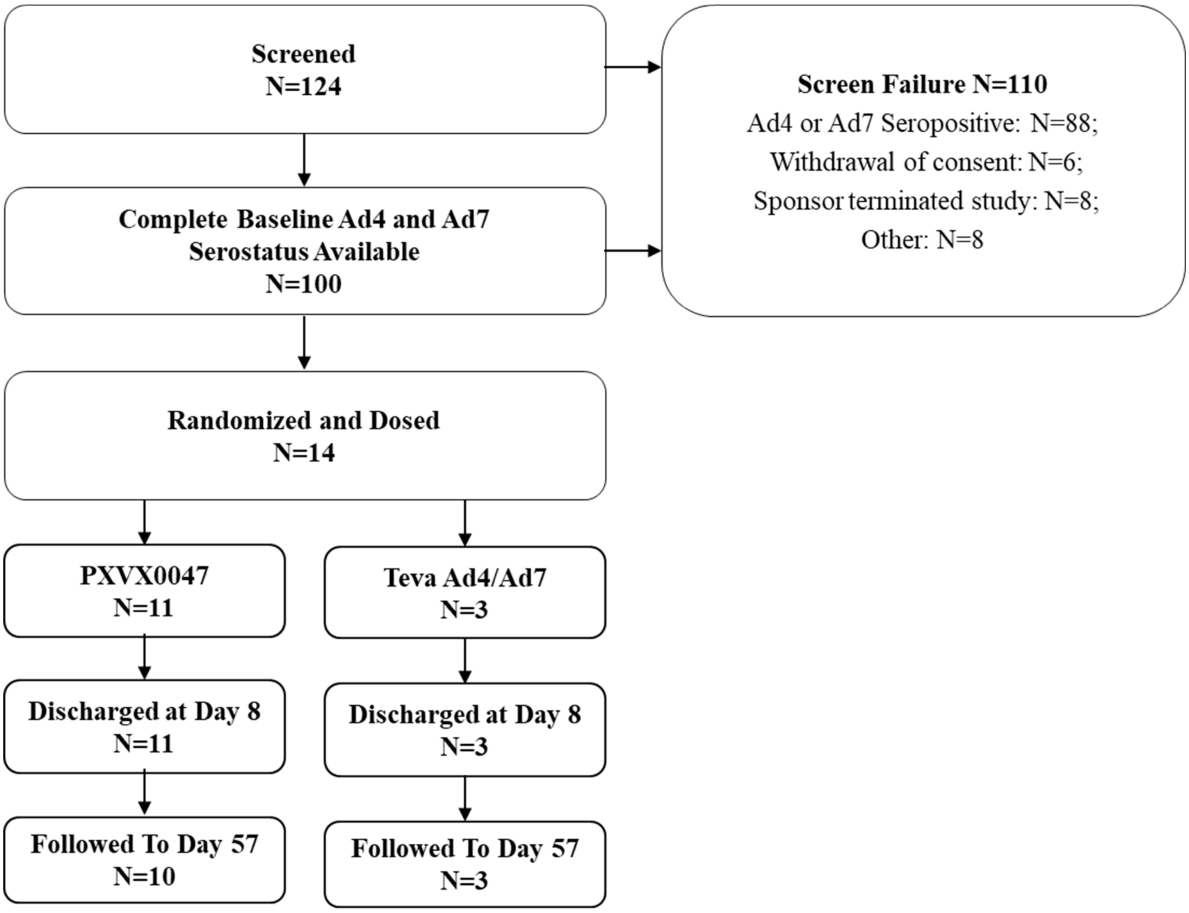

Supplemental Table S1. Treatment-related solicited adverse events by time period

	**Days 1-8**		**Days 8-15**	
**Solicited AE**	PXVX0047 N=11	Teva Ad4/Ad7 N=3	PXVX0047 N=11	Teva Ad4/Ad7 N=3
**Any**	**5 (45.5%)**	**2 (66.7%)**	**3 (27.3%)**	**1 (33.3)**
Nasal congestion	4 (36.4%)	1 (33.1%)	0	0
Headache	3 (27.3%)	0	2 (18.2%)	1 (33.3%)
Nausea	1 (9.1%)	1 (33.1%)	0	0
Sore Throat	1 (9.1%)	1 (33.1%)	1 (9.1%)	0
Vomiting	1 (9.1%)	1 (33.1%)	1 (9.1%)	0
Abdominal Pain	1 (9.1%)	0	0	0
Chills	1 (9.1%)	0	1 (9.1%)	0
Myalgia	1 (9.1%)	0	0	0
Arthralgia	0	0	0	0
Cough	0	0	1 (9.1%)	0
Diarrhea	0	0	1 (9.1%)	0
Dyspnea	0	0	0	0
Fatigue	0	0	0	0
Fever	0	0	0	0

Supplemental Table S2. Seroconversion rates by timepoint, as measured by luciferase assay.

Serotype	Treatment group	Seroconversion by Day, n/N (%)					Cumulative seroconversion rate
		Day 8	Day 15	Day 22	Day 29	Day 57	
Ad4	PXVX0047	0/11 (0)	1/10 (10.0)	4/11 (36.4)	4/11 (36.4)	2/10 (20.0)	4/11 (36.4)
	Teva	0/3 (0)	1/3 (33.3)	1/2 (50.0)	2/3 (66.7)	1/3 (33.3)	2/3 (66.7)
Ad7	PXVX0047	0/11 (0)	9/10 (90.0)	10/11 (90.9)	9/11 (81.8)	7/10 (70.0)	10/11 (90.9)
	Teva	0/3 (0)	2/3 (66.7)	2/2 (100.0)	3/3 (100)	3/3 (100)	3/3 (100.0)

Seroconversion was defined as a 4-fold or greater rise from baseline. At each timepoint, the denominator is the number of subjects for whom assay results are available. The cumulative seroconversion rate includes all subjects who met seroconversion at any post-vaccination timepoint.

Supplemental Table S3. Ad4 and Ad7 serostatus at screening by luciferase assay

	Ad7 +	Ad7 -	Total
**Ad4 +**	27 (23.1%)	13 (11.1%)	40 (34.2%)
**Ad4 -**	42 (35.9%)	35 (29.9%)	77 (65.8%)
**Total**	69 (59.0%)	48 (41.0%)	117 (100%)

Supplemental Table S4. Ad4 and Ad7 serostatus at screening by colorimetric assay

	Ad7 +	Ad7 -	Total
**Ad4 +**	36 (36.0%)	9 (9.0%)	45 (45.0%)
**Ad4 -**	34 (34.0%)	21 (21.0%)	55 (55.0%)
**Total**	70 (70.0%)	30 (30.0%)	100 (100%)

Supplemental Table S5. Neutralizing Antibodies by Timepoint: Geometric Mean Titers

Assay	Treatment Group	Serotype	Day 1	Day 8	Day 15	Day 22	Day 29	Day 57	Peak*
Luciferase	PXVX0047 (N=11)	Ad4	2.5 (2.5-2.5)	2.5 (2.5-2.5)	4.9 (1.7-13.8)	7.7 (2.6-23.4)	6.6 (2.4-18.1)	3.9 (2.1-7.3)	10.5 (2.9-38.4)
		Ad7	2.5 (2.5-2.5)	3.6 (2.6-4.9)	330.4 (64.8-1683.7)	256.1 (66.7-983.8)	158.4 (42.8-585.9)	67.5 (18.7-243.0)	391.9 (90.7-1692.9)
	Teva Ad4/Ad7 (N=3)	Ad4	2.5 (2.5-2.5)	2.5 (2.5-2.5)	6.4 (0.1-355.5)	36.2 (0.0->10000)	28.2 (0.1-9846.0)	12.3 (0.2-742.1)	34.8 (0.0->10000)
		Ad7	2.5 (2.5-2.5)	2.5 (2.5-2.5)	65.3 (0.1->10000)	111.3 (0.2->10000)	204.0 (12.4-3356.0)	92.7 (16.8-512.2)	217.1 (13.8-3403.7)
Colorimetric	PXVX0047 (N=11)	Ad4	2.6 (2.0-3.3)	2.7 (2.0-3.7)	5.3 (2.3-12.3)	8.1 (3.7-18.0)	7.2 (3.2-16.1)	4.7 (2.3-9.6)	10.0 (3.9-25.2)
		Ad7	2.2 (1.9-2.6)	2.6 (1.9-3.4)	105.2 (24.1-459.7)	82.1 (26.5-254.9)	53.8 (19.0-152.0)	28.8 (11.0-75.6)	115.6 (31.3-426.5)
	Teva Ad4/Ad7 (N=3)	Ad4	2.0 (2.0-2.0)	2.6 (1.5-4.7)	10.1 (0.7-139.9)	31.0 (0.0->10000)	24.8 (0.3-1935.0)	16.0 (0.6-462.9)	38.2 (0.5-2719.6)
		Ad7	2.0 (2.0-2.0)	2.0 (2.0-2.0)	37.3 (0.3-4917.0)	54.1 (11.8-248.1)	85.8 (16.8-436.7)	46.3 (7.2-298.2)	97.2 (24.4-387.2)

*Peak is calculated using the highest post-vaccination value for each subject

Supplemental Table S6. Ad4 and Ad7 viral vaccine sequence strains

Vaccine	Adenovirus component	Genbank accession #
Wyeth	Ad4	AY458656
Wyeth	Ad7	AY594256
Teva	Ad4	AY594254
Teva	Ad7	MH910669.1
PXVX0047	Ad4	MN936177
PXVX0047	Ad7	MN936178

Seroconversion was defined as a 4-fold or greater rise from baseline. N/A indicates data not available.

Supplemental Table S7. Ad4 neutralization titers, as measured by luciferase assay.

Treatment group	Subject ID	Day 1	Day 8	Day 15	Day 22	Day 29	Day 57
PXVX0047	35/M	4	4	4	4	4	4
	21/F	4	4	4	18.3	38.9	12.3
	28/M	4	4	4	4	4	4
	23/F	4	4	256	52.4	13.1	4.1
	35/F	4	4	4	4	4	4
	20/F	4	4	4	4	4	4
	20/F	4	4	6.1	17.9	20.4	28.6
	33/M	4	4	4	4	4	4
	23/M	4	4	N/A	326.3	174.2	N/A
	28/M	4	4	4	4	4	4
	25/M	4	4	7.7	4.3	4	4
Teva	22/F	4	4	4	N/A	32.3	10.9
	27/F	4	4	41.3	523	277.3	67.6
	24/M	4	4	4	4	4	4

Seroconversion was defined as a 4-fold or greater rise from baseline. N/A indicates data not available.

Supplemental Table S8. Ad7 neutralization titers, as measured by luciferase assay.

Treatment group	Subject ID	Day 1	Day 8	Day 15	Day 22	Day 29	Day 57
PXVX0047	35/M	4	4.7	2862.5	1330.4	936.7	305.8
	21/F	4	4	588.6	273.3	140.5	53.6
	28/M	4	4	183.8	328.8	321.6	202.9
	23/F	4	4	77.7	30.7	12.8	7.1
	35/F	4	4	4	4	4	4
	20/F	4	4	704.1	1328	1116.1	541.1
	20/F	4	4	203.7	242	171.9	123.6
	33/M	4	4.2	4438.6	2094.3	1040.9	284.8
	23/M	4	5.1	N/A	538.7	273	N/A
	28/M	4	4	4	4	4	4
	25/M	4	4	7.7	4.3	4	4
Teva	22/F	4	4	597.1	N/A	708.6	177.6
	27/F	4	4	4.3	67.6	78.8	45.1
	24/M	4	4	108.6	183.2	152.1	99.5

Seroconversion was defined as a 4-fold or greater rise from baseline. N/A indicates data not available.

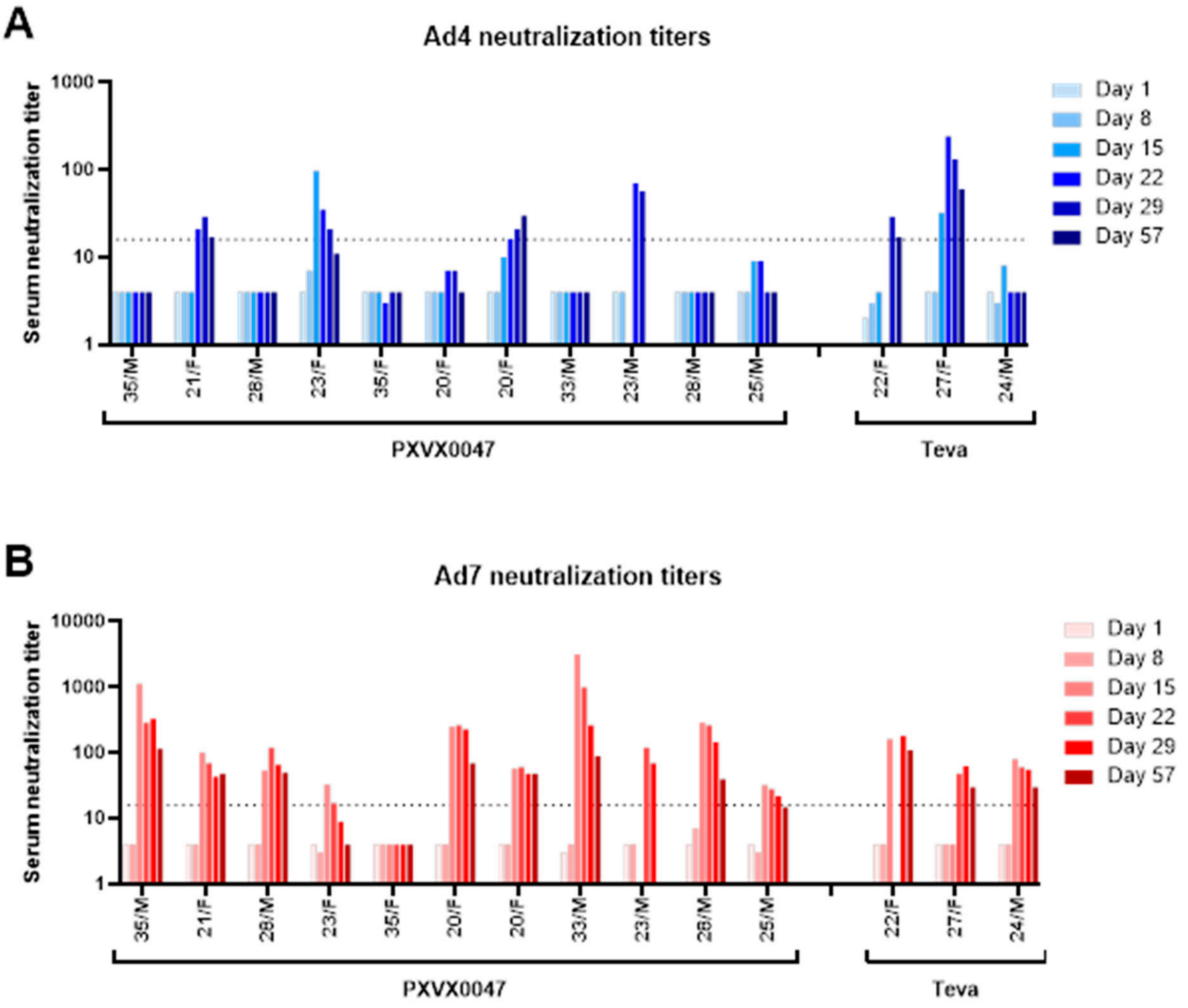

Supplemental Figure S2. Ad7 neutralization titers, as measured by CPE assay.

The age/sex of each study subject is shown on the horizontal axis. Groupings of subjects indicate treatment with PXVX0047 or Teva. The dashed horizontal line indicates the threshold for seroconversion, defined as a 4-fold or greater rise from baseline. Neutralization data is not available for Teva 22/F (Day 22) or 23/M (Days 15 and 57).

Supplemental Table S9. Seroconversion rates by timepoint, as measured by colorimetric assay.

Serotype	Treatment Group	Seroconversion by Day, n/N (%)					Cumulative seroconversion rate
		Day 8	Day 15	Day 22	Day 29	Day 57	
Ad4	PXVX0047	0/11 (0)	1/10 (10.0)	4/11 (36.4)	4/11 (36.4)	2/10 (20.0)	4/11 (36.4)
	Teva	0/3 (0)	1/3 (33.3)	1/2 (50.0)	2/3 (66.7)	2/3 (66.7)	2/3 (66.7)
Ad7	PXVX0047	0/11 (0)	9/10 (90.0)	10/11 (90.9)	9/11 (81.8)	7/10 (70.0)	10/11 (90.9)
	Teva	0/3 (0)	2/3 (66.7)	2/2 (100.0)	3/3 (100)	3/3 (100)	3/3 (100.0)

Seroconversion was defined as a 4-fold or greater rise from baseline. At each timepoint, the denominator is the number of subjects for whom assay results are available. The cumulative seroconversion rate includes all subjects who met seroconversion at any post-vaccination timepoint.
